# Impact of microbiological testing protocols on discard rates in donor human milk with a focus on *Bacillus cereus*

**DOI:** 10.3389/fmicb.2026.1887353

**Published:** 2026-07-29

**Authors:** Vanessa Pleguezuelos, Nerea López, Antonio J. Trujillo, M. Manuela Hernández-Herrero, Gloria Soria

**Affiliations:** 1 Banc de Sang i Teixits (Blood and Tissue Bank of Catalonia), Barcelona, Spain; 2 Transfusion Medicine Research Group, Vall d’Hebron Research Institute (VHIR), Vall d’Hebron University Hospital, Barcelona, Spain; 3 Centre d’Innovació, Recerca i Transferència en Tecnologia dels Aliments (CIRTTA), TECNIO, Departament de Ciència Animal i dels Aliments, Facultat de Veterinària, Universitat Autònoma de Barcelona, Bellaterra, Spain

**Keywords:** *Bacillus cereus*, donor human milk, *Enterobacteriaceae*, human milk, human milk bank, microbiological testing, pasteurization, *Staphylococcus aureus*

## Abstract

Microbiological testing protocols are key components of human milk bank operations, yet the lack of consensus on screening schedules and microbiological criteria leads to variability in discard rates and uncertainty about safety standards. This study provides a large-scale characterization of pre-pasteurization donor human milk microbiota and evaluates the impact of testing protocols on operational outcomes. Over 59 months, 7,921 donor human milk batches were analysed. Pre-pasteurization analyses were dominated by skin staphylococci, with environmental Gram-negative bacilli and enteric genera detected at lower prevalence. Among pathogens of concern, only *Staphylococcus aureus*, *Escherichia coli* and *Bacillus cereus sensu lato* were identified. A selective 24-h workflow, incorporating BACARA® plates with a detection limit of 1 CFU/mL, was validated and implemented to target these organisms within the pre-pasteurization 24-h storage interval. This protocol significantly increased *B. cereus s.l.* detection (2.2% vs. 8.8%), without affecting *S. aureus* or *Enterobacteriaceae* detection, indicating improved analytical sensitivity. *B. cereus s.l.* exhibited a reproducible warm-season excess (5.5% vs. 2.4%) and accounted for 72% of post-pasteurization positives. In a sub-study of 491 batches, agreement between pre- and post-pasteurization *B. cereus s.l.* results was good (*κ* ≈ 0.61) with a high negative predictive value (95%), supporting the predictive value of early screening. Protocol design was a major determinant of discard rates, and using higher microbiological thresholds (2 × 10^5^ CFU/mL) reduced avoidable losses without compromising safety. These findings hightlight the importance of integrating rapid, selective methods and advancing towards harmonized, risk-based microbiological criteria in human milk banking to improve both operational efficiency and microbiological safety.

## 1 Introduction

Human milk is the optimal source of nutrition for infants, providing substantial immunological and developmental benefits, particularly critical for preterm and medically vulnerable newborns (Perrin et al., 2022). When mother’s own milk is unavailable or insufficient to meet the infant’s nutritional requirements, donor human milk (DHM) provided by human milk banks (HMBs) is the recommended alternative (World Health Organization and UNICEF, 2003). Milk banking is currently practiced in 70 countries and represents an established component of neonatal care (Herson and Weaver, 2024).

Human milk often contains a complex microbiota derived from the maternal skin and gastrointestinal tract, as well as microorganisms introduced through retrograde flow from the infant’s oral cavity into the mammary ducts (Demmelmair et al., 2020; Dombrowska-Pali et al., 2024). In addition to this endogenous microbiota, handling during expression, storage, and processing may introduce environmental contamination (Haiden and Ziegler, 2016). Commonly reported genera include *Streptococcus*, *Staphylococcus*, *Micrococcus*, *Lactococcus*, *Lactobacillus*, and *Bifidobacterium* (Dombrowska-Pali et al., 2024). Although transmission of disease via breastfeeding or DHM is uncommon, potentially pathogenic microorganisms such as *Staphylococcus aureus*, *Escherichia coli*, and *Bacillus cereus sensu lato* may be present (Blackshaw et al., 2021; Thayagabalu et al., 2024). This is particularly relevant given the vulnerability of preterm infants, in whom microbial exposure may contribute to severe complications such as late-onset sepsis and necrotizing enterocolitis, supporting the need for strict safety measures in HMBs (Blackshaw et al., 2021; Thayagabalu et al., 2024).

To mitigate microbiological risk, HMBs apply layered controls, including donor eligibility screening, hygienic DHM collection, time–temperature management, microbiological testing, and Holder pasteurization (HoP; 62.5 °C for 30 min), which remains the most widely used processing method (Moro et al., 2019; Weaver et al., 2019; Wesolowska et al., 2019). HoP effectively inactivates vegetative microorganisms while preserving much of the nutritional and immunological value of DHM (Moro et al., 2019; Wesolowska et al., 2019). However, this method is not useful for inactivating bacterial spores, particularly those of *B. cereus s.l.*, which may survive and subsequently germinate under favorable conditions (Kumari and Sarkar, 2016; Liao and Tsai, 2021). This limitation is reflected in evidence from neonatal care settings and HMBs, where *B. cereus s.l.* is a persistent challenge, being frequently associated with post-pasteurization contamination events, microbiological noncompliance, and the discard of DHM (Adjidé et al., 2022; Cormontagne et al., 2021; Jandová et al., 2021).

Nevertheless, human milk exhibits bactericidal activity, contributing to the defense against infections in newborn infants. However, this activity may be compromised when storage exceeds 48 h (Silvestre et al., 2006). In addition, HoP may reduce the intrinsic bactericidal activity of DHM. Therefore, HMB guidelines recommend early freezing, ideally within the first 24 h after expression, in order to preserve its bioactive properties and limit bacterial proliferation (Weaver et al., 2019). Furthermore, thawed DHM stored at 2–8 °C should be processed within 24 h to ensure product quality and safety (Council of Europe, 2022). However, the unavailability of microbiological results for pre-pasteurization milk within this time frame may compel HMBs to proceed with the processing of certain batches in order to comply with established timelines. This situation may result in unnecessary expenditure of human, material and energy resources when these batches are subsequently rejected due to failure to meet the required microbiological criteria.

Regulatory frameworks governing DHM vary internationally and may range from no regulation to classification as a food product or a substance of human origin (Herson and Weaver, 2024). In Europe, Regulation (EU) 2024/1938 defines DHM as a substance of human origin, aligning milk banking practices with tissues and cells for human application and reinforcing requirements for traceability, process validation, and quality assurance (Council of Europe, 2022; European Parliament and the Council of the European Union, 2024).

Despite these advances, operational variability remains substantial across HMBs (Kontopodi et al., 2021). Differences encompass the timing and frequency of microbiological testing, analytical methods, and acceptance thresholds, with direct consequences for rejection rates and the proportion of donations discarded (Clifford et al., 2021; Dewitte et al., 2015; Mullié et al., 2018; Rigourd et al., 2018). Notably, there is still no consensus on microbiological acceptability thresholds for DHM (Kontopodi et al., 2021). Pre-HoP testing practices also differ: the Human Milk Banking Association of North America does not recommend routine testing (Human Milk Banking Association of North America, 2018), whereas in Europe some guidelines propose initial or random testing (Arslanoglu et al., 2023; Calvo et al., 2018; Weaver et al., 2019), and others require testing of every batch (Council of Europe, 2022; National Institute for Health and Care Excellence, 2010). In Sweden and Norway, a portion of DHM is distributed without pasteurization (Grøvslien and Grønn, 2009; Milknet, 2016); In Sweden, DHM must be free of potentially pathogenic bacteria, including *β*-haemolytic streptococci (groups A, C and G), group B streptococci, *Listeria* spp. and *Salmonella* spp., and contain <10^7^ CFU/L of *Staphylococcus aureus*, *Enterobacteriaceae*, *Pseudomonas* spp., *Stenotrophomonas maltophilia* and *Acinetobacter* spp. before dispensing (Milknet, 2016). Across Europe, practices are not uniform because there is not an harmonised protocol yet. A 2021 survey reported that 23% of HMBs tested every individual container and 33% tested every pooled sample of pre-pasteurized DHM (Kontopodi et al., 2021). Foodborne infections linked to expressed human milk, DHM and infant formula have been described even in high income countries, highlighting the importance of rigorous microbiological standards in HMBs (Amenah-James et al., 2025).

Because DHM is a limited and valuable resource, overly stringent protocols may unnecessarily reduce supply and increase costs, whereas overly permissive criteria may compromise safety (Spatz, 2018). Recent reviews and consensus statements from European Milk Bank Association (EMBA) document efforts toward harmonization while recognizing the need for risk-based, context-appropriate criteria (Arslanoglu et al., 2023; Moro et al., 2019; Weaver et al., 2019), and several groups have proposed operational improvements specifically aimed at mitigating *B. cereus s.l.* risk in DHM (Adjidé et al., 2022; Jandová et al., 2021; Mallardi et al., 2022).

This study aimed to analyse the microbiological data from the HMB of Banc de Sang i Teixits (BST), Barcelona, Spain, over 59 months, to characterize profiles in pre- and post-pasteurization DHM and to evaluate the impact of different testing protocols on rejection rates across seasons. This work seeks to provide evidence to contribute to the development of a standardized microbiological quality control protocol for DHM.

## 2 Materials and methods

### 2.1 Study setting and design

This retrospective study was conducted at the HMB of BST. The HMB supplies clinician-prescribed DHM to premature or sick infants when mother’s own milk is unavailable or insufficient and promotes awareness of the value of breastfeeding. Operations follow standardized procedures for donor screening, milk collection, pooling, microbiological quality control, HoP, storage at < −18 °C or < −70 °C, and supply in the frozen state while maintaining the corresponding storage temperature, in accordance with the Council of Europe technical guidelines (Council of Europe, 2022) and EMBA recommendations, as well as spanish national guidance for HMBs (Calvo et al., 2018; Weaver et al., 2019). A convenience sample comprising all available microbiological records generated during the study period was included. Microbiological records from February 2020 to December 2024, inclusive, were extracted from the institutional laboratory database and analyzed.

### 2.2 Donor screening, milk collection, transport and handling

DHM was obtained from approved donors following clinical and social screening criteria and serological testing, in accordance with Royal Decree 1088/2005 (Ministerio de Sanidad y Consumo, 2005). DHM was expressed at home using breast pumps into sterile polypropylene containers (150 mL; Medela S. L., Madrid, Spain) stored at < −18 °C for up to 4 weeks, and collected by the HMB using validated biological transport containers with ice packs and dry ice, maintaining DHM at < −25 °C for up to 8 h during transport.

Upon receipt, DHM was stored at −20 °C and subsequently thawed in a 4 °C cold room for 24 h, ensuring that processing was carried out within 3 months from the date of expression. Aliquots were prepared under aseptic conditions in a Grade A laminar-flow hood (NuAire, Inc., Minnesota, United States) in a grade D classified environment. For HoP, DHM was packaged (50, 100, 250 mL polypropylene containers; Sterifeed, Totnes, United Kingdom), sealed (ICS600, Sterifeed), and pasteurized (S90, Sterifeed). Post-HoP aliquots were prepared following the same aseptic procedure.

### 2.3 Pre-pasteurization microbiological testing

Pre-pasteurization microbiological testing was conducted according to the HMB standard operating procedures using culture media from bioMérieux (Marcy-l’Étoile, France). Two testing protocols were applied over the study period.

During period A (February 2020–September 2023), 5,420 pre-pasteurized DHM samples were analysed using the general microbiological protocol, which consisted of counting and identification of all recovered isolates. A dilution 1:10 was prepared by adding 100 μL of milk to 900 μL of saline solution, followed by a 1:100 dilution prepared by adding 100 μL of 1:10 dilution to 900 μL of saline solution. Aliquots of 100 μL from both dilutions were spread onto sheep blood agar using a sterile Digralsky loop (VWR International, Radnor, PA, United States) and incubated at 36 ± 1 °C for 24–48 h at aerobic conditions. Colonies were counted, and results were expressed as colony-forming units per millilitre (CFU/mL). Morphologically distinct isolates were identified by MatrixAssisted Laser Desorption/Ionization Time-of-Flight (MALDI-TOF; Bruker Daltonics, Bremen, Germany) according to the manufacturer’s protocols. Final reports were obtained 48–72 h after sampling. During this period, quality control was performed simultaneously with DHM pasteurization.

During period B (October 2023–December 2024), 2,501 pre-pasteurized DHM samples were analysed with a selective microbiological protocol. This 24-h selective plating protocol targeting *S. aureus*, *Enterobacteriaceae*, and *B. cereus s.l.* was validated based on the microbiological profile observed during the first 44 months of operation (period A). Dilutions were prepared as described for the general microbiological protocol. Aliquots of 100 μL from the 1:10 and 1:100 dilutions were spread onto selective media: MacConkey agar (MCK) for *Enterobacteriaceae*, Mannitol Salt Agar, also known as Chapman agar (CHA) for *S. aureus*; and non-selective media: Tryptone Soy Agar (TSA) for total aerobic counts. In parallel, 1 mL of milk was plated directly onto BACARA® (BAC) plates to screen for the *B. cereus* group (*B. cereus s.l.*), which includes *B. cereus sensu stricto*, *B. thuringiensis, B. mycoides, B. pseudomycoides, B. anthracis, B. weihenstephanensis*, and *B. cytotoxicus*. After 24-h incubation at 36 °C ± 1 °C at aerobic conditions, plates were examined and colonies counted as follows: TSA, to obtain the total aerobic count (CFU/mL); CHA for the *S. aureus* count (CFU/mL); MCK, to obtain the *Enterobacteriaceae* count (CFU/mL); BAC for the *B. cereus* group (CFU/mL) count. When required, identification was confirmed by MALDI-TOF. Final reports were obtained 24 h after sampling. During this period, quality control was performed first and only acceptable DHM batches were pasteurized.

During both periods, the acceptance criteria, in accordance with NICE recommendations (National Institute for Health and Care Excellence, 2010), were a total aerobic microbial count ≤10^5^ CFU/mL and ≤10^4^ CFU/mL for *S. aureus* and *Enterobacteriaceae*. Moreover, <1 CFU/mL of *B. cereus s.l.* was an additional acceptance criterion at the HMB of BST, given that *B. cereus s.l.* is resistant to HoP.

Seasonality was evaluated using all DHM batches processed between February 2020 and December 2024. Batches were classified into warm and cold periods according to the period in which they were processed and microbiologically analysed. The warm period included batches processed between July and November, corresponding to DHM expressed between May and November. The cold period included batches processed between December and June, corresponding to DHM expressed between October and June.

The frequency of microbiological positivity for *S. aureus*, *Enterobacteriaceae*, and *B. cereus s.l.* was compared between warm and cold periods. In addition, the potential effect of the analytical protocol used (general vs. selective microbiological protocol) and seasonality (warm vs. cold period) on the detection of *B. cereus s.l.* in pre-HoP DHM batches was evaluated.

Microbiological positivity and rejection rates in this study were interpreted according to the acceptance criteria routinely applied by the HMB, as described above.

### 2.4 Post-pasteurization microbiological testing

During period A and B, 6,882 post-pasteurized DHM batches were tested by plating 100 μL onto TSA and incubating at 36 °C ± 1 °C for 24 h in aerobic conditions. Colonies were counted and identified by MALDI-TOF when present. The limit of quantification (LoQ) of this method was 10 CFU/mL. To enhance sensitivity to ≈ 1 CFU/mL and promote germination of *B. cereus s.l.* spores, 1 mL of DHM was enriched in Tryptone Soy Broth (TSB) for 24 h at 36 ± 1 °C, followed by subculture onto TSA and incubation for a further 24 h. Once the sample has been enriched, quantification is no longer possible. Therefore, if the subcultured TSA was positive while the original TSA plate was negative, the microbial bioburden was 1–9 CFU/mL but the exact concentration could not be determined. In this case, it was recorded as 9 CFU/mL for quantitative analysis.

### 2.5 *Bacillus cereus sensu lato* pre-pasteurization and post-pasteurization concordance

A three-month sub-study including 491 DHM samples was performed to assess if presence of *B. cereus s.l*. in pre-HoP DHM could predict its presence in post-HoP DHM. All batches were pasteurized regardless of their initial microbiological results, and concordance between pre- and post-HoP detection of *B. cereus s.l.* was evaluated using Cohen’s kappa coefficient. The predictive performance of pre-HoP screening was assessed by calculating the positive and negative predictive values.

### 2.6 Statistical analysis

Statistical analysis were performed using GraphPad Prism version 5 (GraphPad Software, San Diego, CA, United States). Normality was assessed using the Shapiro–Wilk test. When data were normally distributed, parametric tests were applied; otherwise, log₁₀-transformed values or non-parametric tests were used. Comparisons between two independent groups (period A vs. period B) were performed using the unpaired t-test for normally distributed data or the Mann–Whitney *U* test for skewed distributions. Paired comparisons (pre- vs. post-intervention) were analyzed using the paired *t*-test or the Wilcoxon signed-rank test, as appropriate.

Proportions were reported with 95% confidence intervals. Differences between proportions were assessed using χ^2^ tests or Fisher’s exact tests, as appropriate. Statistical significance was set at *p* < 0.05.

## 3 Results and discussion

### 3.1 Total viable aerobic microbial count of pre-pasteurization donor human milk

**Figure 1:**
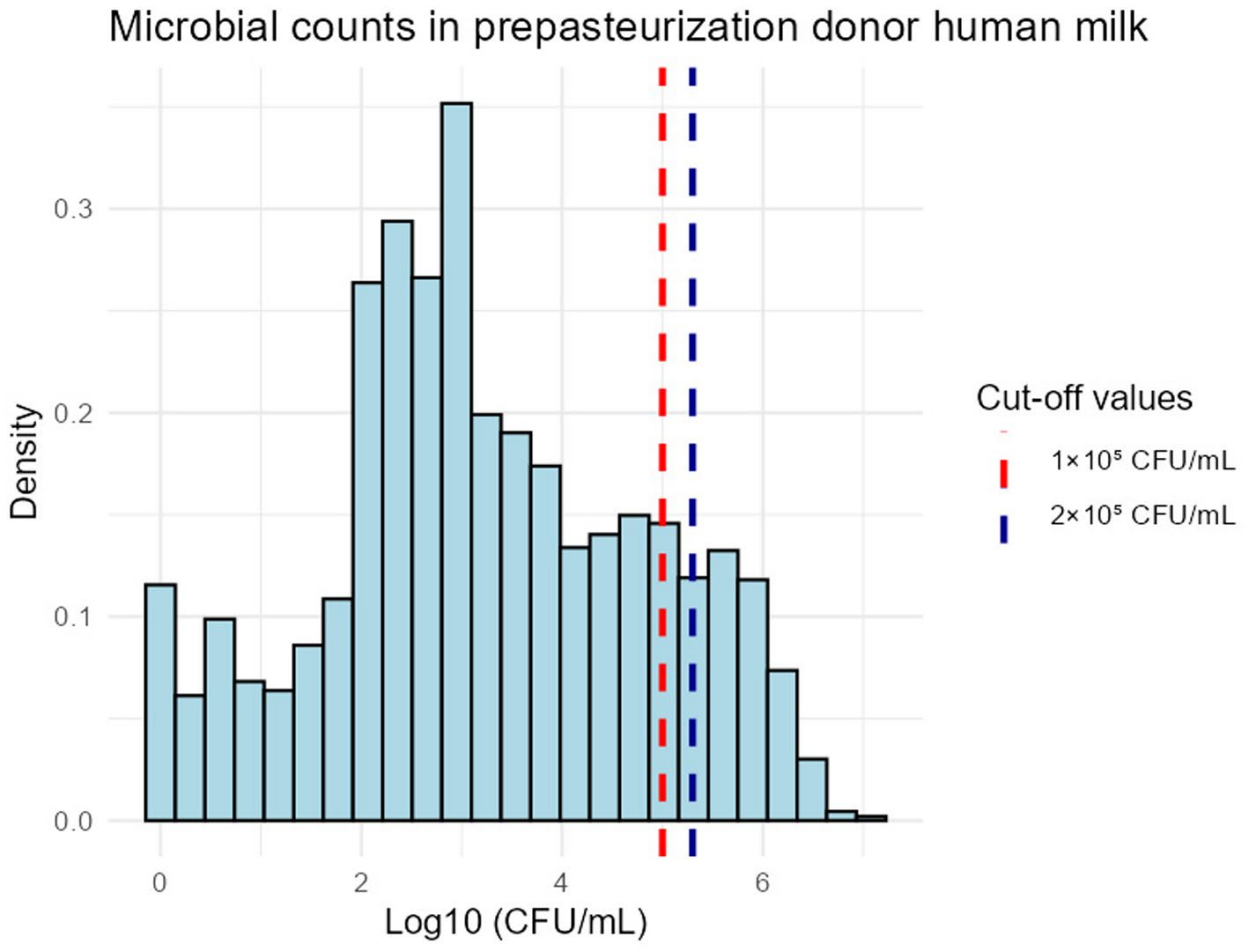
Histogram of total microbial counts in pre-pasteurization donor human milk (log₁₀ CFU/mL) of 7,921 batches analysed between February 2020 and December 2024. The dashed red and blue vertical lines indicate the rejection threshold recommended by the European Milk Bank Association (1 × 10^5^ CFU/mL) and the maximum acceptable count according to the European Pharmacopoeia (2 × 10^5^ CFU/mL), respectively. Pre-pasteurization microbial count in donor human milk by year.

Microbial counts ranged from 0 to 2.0 × 10^7^ CFU/mL (7.3 log₁₀ CFU/mL), with an arithmetic mean of 1.9 × 10^5^ CFU/mL (5.3 log₁₀ CFU/mL) and a median of 4.0 × 10^3^ CFU/mL (3.6 log₁₀ CFU/mL) in 7,921 pre-HoP DHM batches. The interquartile range was 3.0–4.7 log₁₀ CFU/mL, and the 90th percentile reached 5.6 log₁₀ CFU/mL (≈4 × 10^5^ CFU/mL). The global distribution is shown in Figure 1. These findings indicate that most prepasteurized DHM batches exhibited low-to-moderate (<4 log₁₀ CFU/mL) microbial loads, whereas a relatively small number of highly contaminated samples (>5 log₁₀ CFU/mL) disproportionately influenced the upper tail of the distribution. Similar distributions have been reported in previous milk-bank studies describing generally low-to-moderate bacterial loads together with a small fraction of highly contaminated donations that account for most rejections (Almutawif et al., 2017; Miura et al., 2023; Mullié et al., 2018).

Two acceptance thresholds were considered to contextualize pre-HoP total microbial counts in DHM. First, 1 × 10^5^ CFU/mL, which is the reject criterion recommended by the EMBA, Council of Europe Guide to the Quality and Safety of Tissues and Cells for Human Application (Council of Europe, 2022) and adopted by several milkbank guidelines for pre-HoP screening (Arslanoglu et al., 2023; Moro et al., 2019; National Institute for Health and Care Excellence, 2010). Second, 2 × 10^5^ CFU/mL, which was used as an alternative acceptance criterion for microbiological quality based on the European Pharmacopoeia, chapter 2.6.12 (European Directorate for the Quality of Medicines and HealthCare (EDQM), 2023).

**Figure 2:**
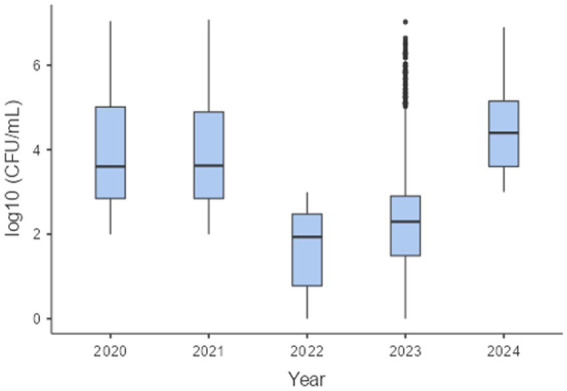
Boxplots of total microbial counts in pre-pasteurization donor human milk by year (log₁₀ CFU/mL). Boxplots show medians (horizontal line), interquartile ranges (boxes), and minimum and maximum values (whiskers). Outliers are represented as individual dots. *Bacillus cereus sensu lato* discard rates by microbiological protocol and season.

Overall, 78.5% of batches were below 1 × 10^5^ CFU/mL and 83.3% were below 2 × 10^5^ CFU/mL. According to the 1 × 10^5^ criterion, 21.5% of batches would be rejected, while applying the 2 × 10^5^ limit reduces rejection to 16.7%. Application of the 1 × 10^5^ CFU/mL threshold recommended by EMBA and the Council of Europe would therefore result in rejection of approximately one-fifth of pre-pasteurized batches, a proportion comparable to that reported in previous studies using the same criterion (Arslanoglu et al., 2023; Weaver et al., 2019). By contrast, adoption of the 2 × 10^5^ CFU/mL limit proposed within the European Pharmacopoeia framework would reduce discards by nearly 5%. These findings illustrate how rejection rates are strongly influenced by the microbiological criteria applied before HoP and by sampling strategies (Clifford et al., 2021; Jandová et al., 2021). Given that HoP is expected to achieve substantial microbial reduction under validated conditions, a limit of 1 × 10^5^ CFU/mL may provide a more conservative buffer against process variability (Arslanoglu et al., 2023; Weaver et al., 2019), whereas a limit of 2 × 10^5^ CFU/mL may reduce that buffer while potentially avoiding unnecessary discards, in line with the European Pharmacopoeia framework European Directorate for the Quality of Medicines and HealthCare (EDQM), 2023. Figure 2 presents boxplots of log-transformed counts by year. No consistent temporal trend was observed, and median values remained stable at approximately 3.6–3.7 log₁₀ CFU/mL throughout the study period. Outliers above 6–7 log₁₀ CFU/mL were present in every year, explaining the persistent tail of the distribution. The stability of microbial quality of the analysed samples over 5 years suggests that donor recruitment, handling, and collection practices remained consistent, although sporadic outliers underscore the need for continued monitoring and robust pre-HoP testing.

### 3.2 Specific microbial count of pre-pasteurization donor human milk

During period A (February 2020–September 2023), all microbial isolates from a total of 5,420 pre-HoP DHM batches were identified by MALDI-TOF. Species-level identification was reported when reliable; otherwise, results are presented at the genus level. Overall, 489 species belonging to 117 genera were found (Supplementary Table S1).

The microbial profile was dominated by *Staphylococcus* spp., detected in 90.34% of batches, with *Staphylococcus epidermidis* as the most prevalent species (73.22%). Environmental Gram-negative bacilli were also frequently identified, including *Pseudomonas* spp. (44.86%), *Acinetobacter* spp. (38.87%), and *Stenotrophomonas* spp. (19.53%). In contrast, enteric genera were less prevalent, such as *Enterobacter* spp. (9.76%), *Klebsiella* spp. (8.22%), and *Serratia* spp. (7.97%). This predominance of skin associated staphylococci, together with a lower prevalence of environmental and enteric Gram-negative genera is consistent with the typical microbiota described in pre-HoP DHM and suggests that milk expression and handling are major sources of contamination (Almutawif et al., 2017; Chang et al., 2013; Hunt et al., 2011; Landers and Updegrove, 2010; Liu et al., 2022; Miura et al., 2023; Strøm et al., 2022; Wang et al., 2023). Yeasts and filamentous fungi were uncommon, being detected only in 1.9% of batches, predominantly *Candida parapsilosis* (1.32%).

Importantly, major foodborne and clinical pathogens relevant to DHM safety including *Listeria monocytogenes*, *Salmonella* spp., *Clostridium perfringens*, *Shigella* spp., *Cronobacter sakazakii*, were not detected in any sample. The absence of these regulated pathogens is reassuring and supports the microbiological safety of DHM collection and handling practices in the studied setting. Among pontentially relevant pathogens, *S. aureus* was identified in 6.43% of batches, whereas *E. coli* (0.82%) and *B. cereus s.l.* (2.33%) were less frequently detected. These organisms exhibited a wide range of concentrations (see Supplementary Table S1), indicating substancial variability in contamination levels across batches. Their detection, even at low prevalence but with wide concentration ranges, highlights the importance of maintaining systematic pre-HoP screening and strict post-HoP criteria (<1 CFU/mL), given their clinical relevance and their role as frequent causes of discards and adverse events in HMBs (Clifford et al., 2021; Cormontagne et al., 2021; Jandová et al., 2021; Lewin et al., 2019; Rigourd et al., 2018).

The frequent detection of environmental microorganisms also highlights the importance of effective preanalytical controls, including time–temperature management, maintenance of cold-chain integrity, and donor education, together with robust microbiological verification procedures to minimise residual risk in the final product (Arslanoglu et al., 2023; Clifford et al., 2021; Weaver et al., 2019).

In this context, a blanket rule requiring rejection based solely on the presence of any pathogen may be excessively conservative and biologically over-inclusive, since many opportunistic microorganisms identified before pasteurization may not represent a meaningful risk at the concentrations observed. For example, although *Pseudomona*s spp. were detected in 44.86% of batches, the clinically relevant species *Pseudomonas aeruginosa* was identified in only 3.80% of samples. Risk-based microbiological criteria may therefore be more appropriate, as they better reflect both process capability and infant safety. HoP effectively inactivates vegetative bacterial cells but does not neutralise heat-stable toxins. Consequently, the principal concern is substantial pre-HoP growth of toxin-producing microorganisms. Detectable heat-stable enterotoxins are generally associated with bacterial loads around 10^5^ CFU/mL for *S. aureus and B. cereus s.l*. (Fujikawa and Morozumi, 2006; Lewin et al., 2019; Rigourd et al., 2018; Walker-York-Moore et al., 2017), whereas the clinical significance of heat-stable Gram-negative endotoxins and their potential association with necrotising enterocolitis remains under debate (Duess et al., 2023).

Aligned with this risk-based approach, a selective-plating protocol for pre-HoP DHM that focuses on *S. aureus*, *Enterobacteriaceae* and *B. cereus s.l*. was developed at BST and used in Period B.

### 3.3 Pathogen prevalence in pre-pasteurization donor human milk: methodological and seasonal determinants

Regarding the prevalence of pathogens detected in pre-HoP DHM during the period A (general protocol) and B (selective protocol), significant differences were observed in the detection of *B. cereus s.l.*, with prevalences of 2.2% in the first period and 8.8% in the second whereas detection frequencies for *S. aureus* and *Enterobacteriaceae* did not differ significantly (Table 1).

**Table 1:** Prevalence of pathogens in pre-pasteurization donor human milk by analytical protocol (Period A: 444 February 2020–September 2023, general protocol, *n* = 5,420; Period B: October 2023–December 2024, selective protocol, *n* = 2,501).

Microorganisms	Period A	95% CI	Period B	95% CI	p-value
*Staphylococcus aureus* >10^4^ CFU/mL	2.3	(1.8–2.9)	1.5	(0.2–2.9)	0.2593
*Enterobacteriaceae* >10^4^ CFU/mL	10.9	(9.5–12.2)	10.9	(9.6–12.1)	0.9779
*Bacillus cereus sensu lato* ≥1 CFU/mL	2.2	(1.3–2.7)	8.8	(6.4–11.1)	< 0.0001

Values are expressed as a percentage of positive batches (%) with 95% confidence intervals (CI). Period A: general protocol, identification of all isolates (*n* = 5,420). Period B: selective plating protocol (*n* = 2,501). Rejection thresholds: *Staphylococcus aureus* and *Enterobacteriaceae* >10⁴ CFU/mL; *Bacillus cereus sensu lato* ≥1 CFU/mL (limit of detection: Period A = 10 CFU/mL; Period B = 1 CFU/mL).

Evaluation of *B. cereus s.l.* concentration distributions showed that between-protocol differences were confined to categories <100 CFU/mL, with no significant differences for categories ≥100 CFU/mL (Table 2). This pattern is consistent with methodological characteristics: the BACARA® chromogenic medium used in Period B enables detection of 1 CFU/mL, whereas the general microbiological protocol used in Period A has a 10 CFU/mL limit of detection. BACARA® medium had been validated for the enumeration of the *B. cereus* s.l. in food matrices, including cow milk (Tallent et al., 2012). This medium has been reported to offer higher sensitivity and specificity than traditional Mannitol Egg Yolk Polymyxin (iMYP) agar, in line with reports that chromogenic media outperform conventional agar for this purpose (Fuchs et al., 2022). These findings indicate that the higher prevalence observed during period B primarily reflects enhanced analytical sensitivity rather than a deterioration in the microbiological quality of DHM (Fuchs et al., 2022). Given the ubiquity of *B. cereus* s.l. in the environment, increased analytical sensitivity may consequently lead to higher rejection rates (Lewin et al., 2019).

**Table 2:** Distribution of *Bacillus cereus sensu lato* counts in pre-pasteurization donor human milk by analytical protocol (Period A: February 2020–September 2023, general protocol, *n* = 5,420; Period B: October 2023–December 2024, selective protocol, *n* = 2,501).

*Bacillus cereus sensu lato* (CFU/mL)	Period A	95% CI	Period B	95% CI	*p*-value
0	98.05	(97.37–98.72)	91.21	(88.88–93.54)	<0.0001
1–9	0.03	(0.00–0.10)	4.99	(3.66–6.32)	<0.0001
10–99	0.05	(0.00–0.11)	2.05	(1.34–2.78)	<0.0001
100–999	0.69	(0.39–0.99)	0.88	(0.40–1.37)	0.1879
≥1,000	1.22	(0.68–1.77)	0.87	(0.26–1.47)	0.8665

Values are expressed as a percentage of positive batches (%) with 95% confidence intervals (CI). Period A: general protocol, identification of all isolates (*n* = 5,420). Period B: selective plating protocol (*n* = 2,501). Limit of detection: Period A = 10 CFU/mL; Period B = 1 CFU/mL.

Seasonality was assessed by comparing warm and cold periods across February 2020–December 2024 (Table 3). Warm months were defined as batches processed and microbiologically tested between July and November (expression date May–November), whereas cold months corresponded to batches processed and tested between December and June (expression date October–June). No statistically significant seasonal variation was observed for *S. aureus* or *Enterobacteriaceae*, as the warm and cold estimates overlapped. In contrast, *B. cereus s.l.* showed a clear warm-season excess (5.5% vs. 2.4%, *p* < 0.0001; Table 3). Similar seasonal increases in *B. cereus s.l.* have been reported in HMBs (Jandová et al., 2021). Furthermore, both analytical protocol (general vs. selective) and season (warm vs. cold) were independently associated with the percentage of pre-HoP DHM batches positive for *B. cereus s.l.* (*p* < 0.0001 for each factor), indicating that environmental conditions and method sensitivity contribute additively.

**Table 3:** Prevalence (%) of pathogens in pre-pasteurization donor human milk by season.

Microorganisms	Warm	95% CI	Cold	95% CI	*p*-value
*Staphylococcus aureus* >10^4^ CFU/mL	2.1	(1.1–3.1)	2.1	(1.5–2.7)	0.9668
*Enterobacteriaceae* >10^4^ CFU/mL	11.5	(10.1–13.0)	10.4	(8.9–11.9)	0.2935
*Bacillus cereus sensu lato* ≥1 CFU/mL	5.5	(3.4–7.6)	2.4	(1.5–3.4)	<0.0001

Values are percentages of positive batches (%) with 95% confidence intervals (CI). Warm: July–November. Cold: December–June. Rejection thresholds: *Staphylococcus aureus* and *Enterobacteriaceae*, >10⁴ CFU/mL; *Bacillus sensu lato*, ≥ 1 CFU/mL.

### 3.4 Pre-pasteurization donor human milk rejection rates

Between February 2020 and December 2024, 7,921 DHM batches were tested, of which 2,267 (28.6%) failed to meet the established microbiological criteria. Overall, 21.9% of batches showed total counts above 10^5^ CFU/mL, making this the main cause of rejection and accounting for 76.5% of all discarded batches (Tables 4, 5). Elevated levels of *Enterobacteriaceae* or *S. aureus* were frequently associated with >10^5^ CFU/mL counts; only 2.7 and 0.6% of batches, respectively, were discarded exclusively due to these targets. Overall, *Enterobacteriaceae* (9.6%), and *S. aureus* (2.0%) contributed a lower rejection rate.

**Table 4:** Donor human milk batches meeting rejection thresholds, expressed % of all batches (*N* = 7,921).

Criteria	Percentage of rejection (%)
Total count >10^5^ CFU/mL	21.9
*Staphylococcus aureus* >10^4^ CFU/mL	1.9
*Staphylococcus aureus* >10^4^ CFU/mL & Total count <10^5^ CFU/mL	0.6
*Enterobacteriaceae* >10^4^ CFU/mL	11.0
*Enterobacteriaceae* >10^4^ CFU/mL & Total count <10^5^ CFU/mL	2.7
*Bacillus cereus sensu lato* ≥1 CFU/mL	4.3
*Bacillus cereus sensu lato* ≥1 CFU/mL & Total count <10^5^ CFU/mL	3.1

**Table 5:** Number and percentage of pre-pasteurization donor human milk (DHM) batches discarded due to microbiological criteria (*N* = 7,921).

Rejection motives	*N* (%)	Discarded DHM (%)
Total count >10^5^ CFU/mL	1,734 (21.9)	76.5
*Enterobacteriaceae* >10^4^ CFU/mL & Total count <10^5^ CFU/mL	218 (2.7)	9.6
*Staphylococcus aureus* >10^4^ CFU/mL & Total count <10^5^ CFU/mL	46 (0.6)	2.0
*Bacillus cereus sensu lato* ≥1 CFU/mL & Total count <10^5^ CFU/mL	242 (3.1)	10.7
Other* & Total count < 10^5^ CFU/mL	27 (0.3)	1.2
Total discarded DHM	2,267 (28.6)	100

The relative contribution of each rejection motive is expressed as a percentage of the discarded batches.

^*^The combinations of two pathogens: *Staphylococcus aureus + Enterobacteriaceae, Staphylococcus aureus + Bacillus cereus sensu lato* or *Enterobacteriaceae + Bacillus cereus sensu lato* are included.

The presence of ≥1 CFU/mL of *B. cereus s.l.* was detected in 4.3% of batches, and in 3.1% of cases this was the sole rejection criterion (Table 4). Among discarded batches, *B. cereus s.l.* ≥1 CFU/mL represented the second most frequent reason of discard, accounting for 10.7% of all rejections (Table 5). This prevalence is higher than prior reports, as *Bacillaceae* were detected in 1.5% of pre-HoP samples by Lindemann *et al*. Lindemann et al. (2004) and in 5% by Landers and Updegrove (2010), probably due to the higher sensitivity of BST selective protocol.

**Figure 3:**
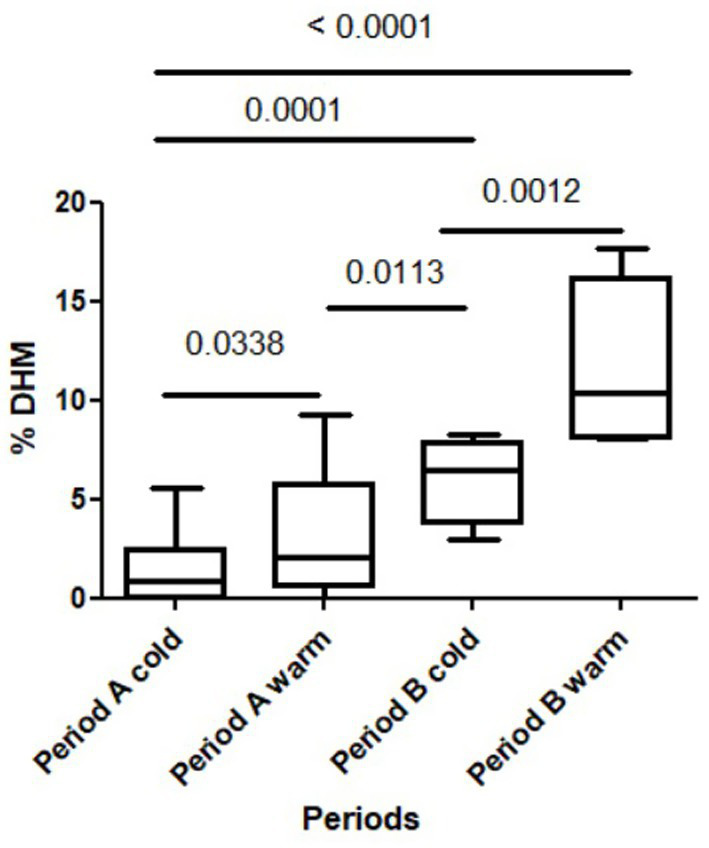
Discard rates (% DHM batches with ≥1 CFU/mL *Bacillus cereus sensu lato*) by microbiological protocol (Period A: identification of all isolates; Period B: selective plating) and season (Cold = December–June; Warm = July–November). Boxes represent the interquartile range; the line within the box indicates the median. Whiskers denote the minimum and maximum values. *p*-values for selected comparisons are indicated.

As shown in Figure 3, *B. cereus s.l.* was the only target for which discard rates varied significantly according to both protocol and season. The higher discard rates observed during warm months are consistent with the well-established capacity of this spore-forming bacterium to grow and persist under favourable environmental conditions. In addition, the selective protocol, which detects levels as low as 1 CFU/mL, revealed a greater proportion of positive batches than the general microbiological protocol. This highlights the relevance of detection methodology in assessing DHM safety and indicates that *B. cereus s.l.* may represent a distinct and policy-sensitive rejection criterion, independent of overall microbial load, although, notably, no standards specifically address the presence of *B. cereus s.l*. yet.

The selective microbiological protocol used in Period B offers a more favourable cost–benefit profile: it is less time-consuming, requires fewer resources, and provides results within 24 h, which is the recommended maximum refrigeration time before HoP (Council of Europe, 2022). Thus, results can be used prospectively to decide which DHM should be pasteurized. By contrast, the general microbiological protocol used in period A based on the enumeration and identification of all microbial isolates typically require 48–72 h to generate results. This situation leads to the processing of batches that are subsequently rejected for failing to meet the established microbiological criteria, resulting in unnecessary consumption of human, material, and energy resources.

### 3.5 Post-pasteurization donor human milk

From February 2020 to December 2024, 6.3% of post-HoP DHM batches (431 out of 6,882) showed microbial counts ≥1 CFU/mL, which is the HMB’s internal rejection threshold. Among positives, counts ranged from 1 to 2 × 10^4^ CFU/mL (median 20 CFU/mL; mean 300 CFU/mL). Notably, 18.3% of positive batches were <10 CFU/mL. Most positive cultures involved spore-forming bacteria (94%), and *B. cereus s.l.* was isolated in 72% of the positive batches. Importantly, rejection rates due to *B. cereus s.l.* did not differ between pre-HoP analytical protocols (general vs. selective; *p* = 0.1080). However, discard rates were slightly higher in warm months than in cold months (7.2% vs. 4.8%; *p* = 0.0497). This seasonal effect is consistent with the pre-HoP results, where only *B. cereus s.l.* showed a marked increase during warm months.

In post-HoP samples with microbial growth, as evaluated by other authors, *Bacillus* is present in approximately 60–74% of samples, with *B. cereus s.l.* being the most frequently identified group (Adjidé et al., 2022; Padín Fontán et al., 2022). Discards attributed to *B. cereus s.l.* represent a relevant proportion of rejected batches in HMBs, although reported rates vary according to local practices and the microbiological methods employed (Billeaud, 2021; Landers and Updegrove, 2010). A seasonal effect has also been reported, with higher detection rates during warmer months (Adjidé et al., 2022).

Criteria for post-HoP testing remain inconsistent: some sources advise testing all batches (Moro et al., 2019; National Institute for Health and Care Excellence, 2010), while others do not mandate routine testing (Arslanoglu et al., 2023). Acceptance thresholds also diverge, with several guidelines tolerating <10 CFU/mL (Arslanoglu et al., 2023; Calvo et al., 2018; Tran et al., 2023), whereas others mandate no growth (Council of Europe, 2022; Moro et al., 2019; National Institute for Health and Care Excellence, 2010; Weaver et al., 2019). In Europe, Kotopodi’s survey (Kontopodi et al., 2021) found that only 56% of HMBs always test after HoP; 62% accept only no detected growth, 13% accept <10 CFU/mL, 8% accept <100 CFU/mL or have no threshold, and 17% either do not pasteurize or do not test. At HMB of BST, every batch is tested and no growth is accepted.

### 3.6 *Bacillus cereus sensu lato* in pre- and post-pasteurization donor human milk

The concordance between the detection of *B. cereus s.l.* in pre- and post- HoP was evaluated. *B. cereus s.l.* was not detected in both pre- and post-HoP in 419 batches (85.3%). In 35 batches (7.1%), the microorganism was detected both before and after HoP, with a mean pre-treatment count of 3,026 CFU/mL (median 120, range 1–40,000). In 14 batches (2.9%), *B. cereus s.l.* was detected pre-HoP but not recovered post-HoP; these cases had very low initial levels (mean 3.7 CFU/mL; median 1; range 1–13), consistent with inactivation of vegetative cells during HoP. Conversely, 23 batches (4.7%) were negative pre-HoP but positive post-HoP, most often at low levels (<10 CFU/mL in 82.6% of cases), a pattern compatible with the germination of dormant spores into vegetative cells after HoP.

The overall agreement between pre- and post-HoP detection was 92.5%, with a Cohen’s kappa of 0.612 (95% CI: 0.504–0.700), indicating good concordance. The positive and negative predictive values of BACARA® plates were 71.4 and 94.8%, respectively. These data indicate that the selective detection of *B. cereus s.l.* in pre-HoP DHM is a reliable predictor of post-HoP positivity, particularly at counts exceeding 10 CFU/mL. Applying a rejection criterion of any detectable growth (>0 CFU/mL) at the pre-HoP stage prevents the processing of batches that are likely to test positive after HoP. This is the protocol implemented at BST.

### 3.7 Global donor human milk discard rate

**Figure 4:**
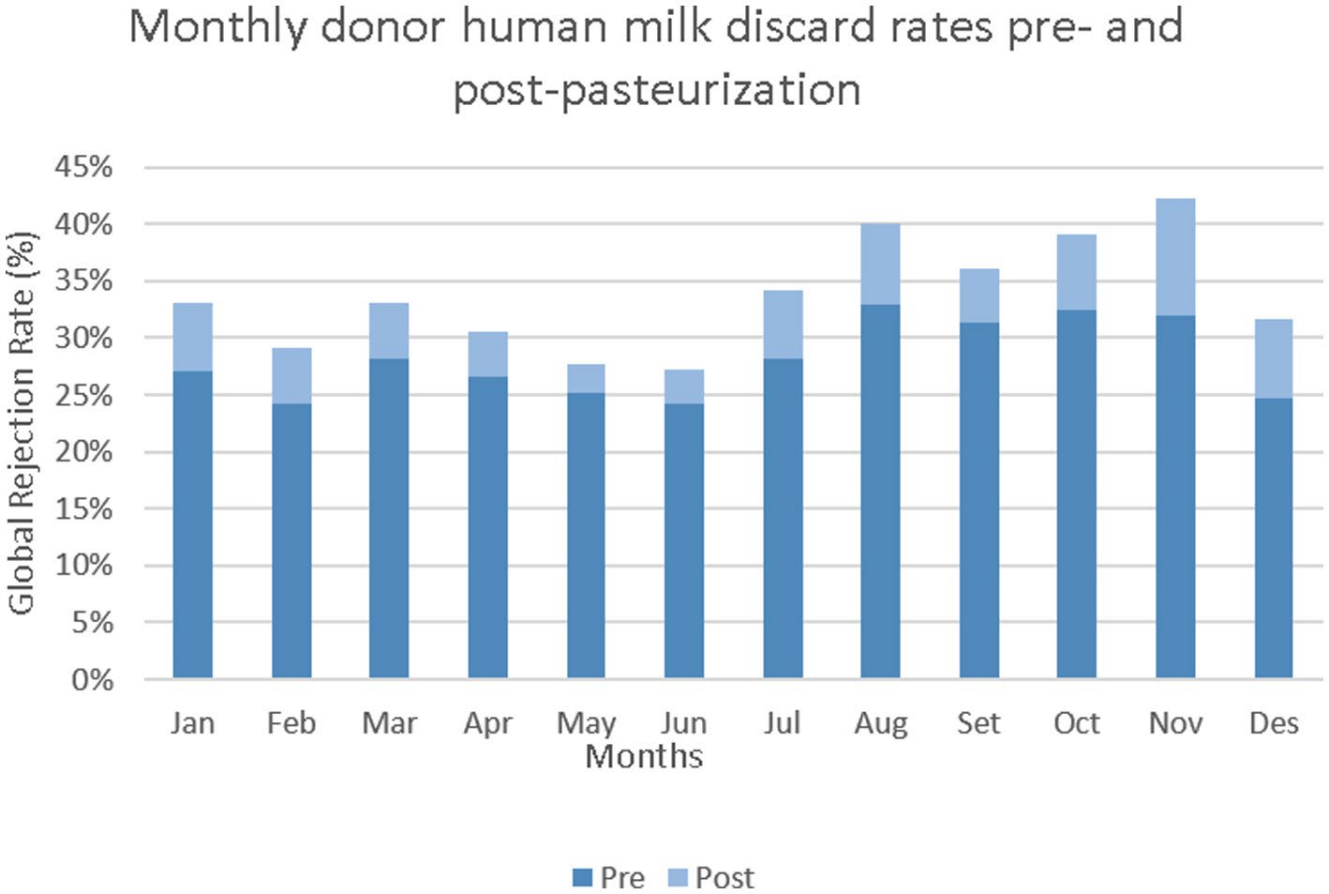
Donor human milk batches discarded after pre- or post-pasteurization microbiological testing. Monthly averages (Feb 2020–Dec 2024, *N* = 7,921); warm months defined as July–November.

Considering both pre- and post-HoP, the overall discard rate was 34.5% (95% CI, 32.2–36.9). Rates did not differ between microbiological protocols in period A vs. B (*p* = 0.7474) but were higher in warm months than in cold months (38.6% vs. 31.5%). The monthly rejection rate of DHM showed a clear increase in the warm season (Figure 4). From December to June, the global rejection rate remained lower (≈27–33%), whereas from July to November it rose progressively, peaking in November at ≈44%, and declining again in December (≈32%). In all months, most rejections occurred at the pre-HoP stage, with a small contribution due to the post-HoP rejection.

Reported overall rejection rates vary widely in the literature, ranging from 5.8% when no pre-HoP screening is performed (Froh et al., 2018) to 27.3% when both pre- and post-HoP controls are applied (Adjidé et al., 2022).

In the present study, overall rejection rates are higher than those reported by other authors, likely because a more sensitive detection strategy is applied before and after HoP. Before HoP, *B. cereus s.l.* is detected using selective plating; after HoP, a liquid enrichment medium is used to allow spores to germinate.

Rigourd et al. (2018) also reported that adding enrichment to the post-HoP protocol doubled the rejection rate. From a risk perspective, attention to *B. cereus s.l.* at low counts is justified because spores can survive HoP and later germinate, and toxigenic potential is strain dependent. Lewin et al. (2019) support a quantitative, risk-based approach to post-HoP control. Rapid molecular assays to detect *B. cereus s.l.* are being developed (Aran et al., 2025).

Further investigation is needed to determine whether aspects of milk collection can be modified to reduce the presence of *B. cereus s.l*. Reductions in post-HoP noncompliance due to *B. cereus s.l.* after corrective actions have been reported (Adjidé et al., 2022; Mullié et al., 2018).

Moreover, there is an increasing interest in obtaining DHM that remains safe while preserving its bioactivity, with particular emphasis on reducing or eliminating discards attributable to total viable counts, pathogens, or *B. cereus s.l.* through more effective analytical and processing strategies. Process innovations such as High Temperature Short-Time pasteurization, High Hydrostatic Pressure or Ultra-High-Pressure Homogenization are being explored to better preserve heat-labile components; however, they must be evaluated against rigorous microbiological performance standards before widespread adoption (Billeaud, 2021; Escuder-Vieco et al., 2018; Jalali et al., 2025; Wesolowska et al., 2019). Until such evidence is consolidated, HoP remains the benchmark in most HMBs (Moro et al., 2019; Weaver et al., 2019).

## 4 Conclusion

This work identified first that the only pathogens of clinical relevance in pre-pasteurized DHM were *S. aureus*, *Enterobacteriaceae*, and *B. cereus s.l*. Subsequently, the implementation of a rapid selective-plating based microbiological protocol focusing on these pathogens showed improved sensitivity to detect *B. cereus s.l.* and enabled microbiological results to be obtained within the 24-h pre-pasteurization storage interval. Therefore, the microbiological results could be used prospectively to decide which DHM should be pasteurized, avoiding the unnecessary consumption of human, material, and energy resources.

This work showed that particular attention should be paid to *B. cereus s.l.* In pre-HoP controls, 3.1% of DHM samples did not meet the acceptance criterion of *B. cereus s.l.* <1 CFU/mL, thus being the second most frequent cause of rejection, only after microbial count >10^5^ CFU/mL, which was the first contributor to rejection in 21.9% of DHM. The data indicate that the selective detection of *B. cereus s.l.* in pre-HoP DHM is a reliable predictor of post-HoP positivity and that applying a rejection criterion of any detectable growth (>0 CFU/mL) at the pre-HoP stage prevents the processing of batches that are likely to test positive after HoP. Even with this criterion in place, in failed post-HoP controls (6.3% of post-HoP DHM batches), *B. cereus s.l.* was the most frequently found microorganism, isolated in 72% of failed batches. Finally, it has to be noted that DHM rejection due to *B. cereus s.l.* was higher in warm months, both at pre-HoP and post-HoP stages.

These findings highlight that microbiological testing protocols are not merely technical procedures, but key factors influencing both the availability and microbiological safety of DHM. Progress towards harmonized, riskbased microbiological criteria for both pre- and post-pasteurization stages, with specific criteria for *B. cereus s.l*., is warranted. Such an approach would help align analytical sensitivity with clinically meaningful risk thresholds, promote greater consistency among HMBs, and optimize the balance between microbiological safety and DHM availability.

## Data Availability

The original contributions presented in the study are included in the article/Supplementary material, further inquiries can be directed to the corresponding authors.
